# Classification of Human Motions Using Micro-Doppler Radar in the Environments with Micro-Motion Interference

**DOI:** 10.3390/s19112598

**Published:** 2019-06-07

**Authors:** Xiaolin Ma, Running Zhao, Xinhua Liu, Hailan Kuang, Mohammed A. A. Al-qaness

**Affiliations:** 1 Key Laboratory of Fiber Optical Sensing Technology and Information Processing, Ministry of Education, and Hubei Key Laboratory of Broadband Wireless Communication and Sensor Networks, School of Information Engineering, Wuhan University of Technology, Wuhan 430070, China; zhaorunning@whut.edu.cn (R.Z.); liuxinhua@whut.edu.cn (X.L.); kuanghailan@whut.edu.cn (H.K.); 2 School of Computer Science, Wuhan University, Wuhan 430072, China; alqaness@whu.edu.cn

**Keywords:** human motion classification, non-target micro-motion interference, micro-Doppler effect, continuous wave radar

## Abstract

Human motion classification based on micro-Doppler effect has been widely used in various fields. However, the motion classification performance would be greatly degraded if the wireless environment has non-target micro-motion interference. In this case, the interference signal aliases with the signal of target human motions and then generates cross-terms, making the signals hard to be used to identify target human motions. Existing methods do not consider this non-target micro-motion interference and have poor resistance to such interference. In this paper, we propose a target human motion classification system that can work in the scenarios with non-target micro-motion interference. Specifically, we build a continuous wave radar transceiver working in a low-frequency radar band using the software defined radio equipment Universal Software Radio Peripheral (USRP) N210 to collect signals. Moreover, we use Empirical Mode Decomposition and S-transform successively to remove non-target micro-motion interference and improve the time-frequency resolution of the raw signal. Then, an Energy Aggregation method based on S-method is proposed, which can suppress cross-terms and background noise. Furthermore, we extract a set of features and classify four human motions by adopting Bagged Trees. Extensive experiments using the test-bed show that under the scenarios with non-target micro-motion interference, 97.3% classification accuracy can be achieved.

## 1. Introduction

Human motion detection and classification systems have been widely used due to the increased demands on social security and surveillance systems. These systems can be implemented by various techniques [1], such as image-based or video-based techniques, and wireless radar-based techniques. Compared to image-based or video-based techniques, radar-based human motion detection and classification systems can work in scenarios regardless of light, in smoke and dust conditions, and even more, some have through-wall capabilities [2,3].

Human motion can cause frequency shift of a radar echo signal, and then produce corresponding Doppler signatures. Thus, Doppler radars can be used to detect human motion. Chen et al. [4] proposed the concept that an object or any structures on the object may have mechanical vibrations or rotations, called micro-motion dynamics. Micro-motion dynamics may induce additional frequency modulations on the returned radar signal, which generate sidebands about the target’s Doppler frequency, called the micro-Doppler effect. A micro-Doppler signal can be regarded as a unique signature of a motion aroused by the human body. Therefore, it can be applied to human motion classification [5,6,7,8,9,10,11,12]. However, as the complexity of the applied environment increases, non-target (i.e., not the target human body) micro-motion interference may appear. Such an interference signal can alias with the signal of the target human motion, and then generate cross-terms, which means the collected signals cannot reflect the real body motion of the target human. Then, systems applied in such scenarios would be much more complex than those for single human body scenarios without any micro-motion interference.

Fioranelli et al. [13] used multiple sets of X-band continuous wave radars to collect the signal associated with human motion. Then, they extracted features based on Short-Time Fourier Transform (STFT) and classified the unarmed and potentially armed personnel. The experimental results showed that the three-node multistatic system has higher classification accuracy than that of the single monostatic radar. In a previous study [14], researchers used the Frequency Modulated Continuous Wave (FMCW) radar system with a carrier frequency of 77 GHz to analyze the human micro-Doppler signatures of one or two persons. In a previous study [15], authors used a continuous wave radar system with a carrier frequency of 24 GHz to detect three types of human motions: two-arm motion, one-arm motion, and no-arm motion. Based on the spectrogram obtained by STFT, they extracted motion features for classification, in which the accuracy is 91.9%.

The aforementioned studies have high requirements on the equipment, and most of them use high-frequency radar band or multistatic radars. As the radar frequency increases, the resolution will increase, and the resolution of the time-frequency distribution obtained by time-frequency analysis will also increase. In this case, it is easier to distinguish the motion state of single or multiple people. Additionally, increasing the number of radars can result in richer echo signals of moving human bodies, so better classification performance can be obtained. However, both increasing the carrier frequency of radar and increasing the number of radars require high costs on hardware. On the other hand, some works [16,17,18,19,20,21] focus on feature extraction. Under the premise of reducing the radar frequency appropriately, the accuracy of motion states classification can be ensured by selecting proper features or adopting effective feature extraction methods. Fairchild et al. [16] used S-band radar and W-band millimeter wave radar to identify four types of motions, namely human walking, standing, bending, and swinging, in cases of through-wall and non-through-wall environments. In the work, the Empirical Mode Decomposition (EMD) was used to produce a unique feature vector from the human micro-Doppler signals, following which a Support Vector Machine (SVM) was used to classify human motions, and the accuracy rates were 75% and 90%, respectively. Also, there are many other methods [17,18,19,20] that can be used in human motion classification, such as Linear Predictive Coding (LPC), Singular Value Decomposition, and Hierarchical Image Classification Architecture. In a previous study [21], researchers used Information-Theoretic (IT) feature selection techniques to identify essential features and minimize the total number of required features, while maximizing classification performance. Results show that for signal-noise-ratios over 10 dB and at least 1 s of data, this approach yields 96% correct classification.

However, the echo signals of the human body acquired in the low-frequency radar band have low time-frequency aggregation and resolution, and high classification accuracy cannot be guaranteed by the improvement of feature extraction only. Therefore, some researchers improved the aggregation degree in the time-frequency domain through time-frequency processing and improved the resolution of the time-frequency distribution to facilitate feature extraction for subsequent classification procedures. For instance, a Hermite multi-window algorithm based on S-method was proposed in previous studies [22,23], which used a few first orders of Hermite functions to improve time-frequency aggregation. Then, they extracted features to classify human gait. In a previous study [24], the authors used the multiwindow Adaptive S-method (AS-method) to analyze radar echoes collected in indoor and outdoor non-interference environments. They separated six states of human motions using the SVM classifier trained by the extracted features. The trained SVM can detect a human body with an accuracy of 95.4% for the two cases without interference. Although these methods can classify human motions under the low-frequency radar band using the time-frequency analysis, all of the signals were collected in the environments without non-target interference. If micro-motion interference aroused by a non-target human body exists, the interference signal would alias with the signal of target human motion and then generate cross-terms. The existing studies cannot effectively resist such interference or reduce its influence on the signal of the target human motion. Therefore, the time-frequency distribution after the time-frequency processing cannot reflect the real body motion of the target human, and misjudgment is likely to occur at the procedure of classification. 

In this paper, we propose a target human motion classification system working in the low-frequency radar band, suitable for non-target micro-motion interference, which can achieve accurate classification of the target human motions in the scenarios with the interference from non-target micro-motion. Particularly, we build a continuous wave radar transceiver with a carrier frequency of 4.2 GHz for radar echo data acquisition of human motion in cases of existing non-target micro-motion interference. In the non-target micro-motion interference scenarios, the interference signal aliases with the signal of target human motion and then generates cross-terms. In order to achieve accurate classification of human motion under low-frequency radar bands in the case of micro-motion interference from non-targets, we design a time-frequency analysis scheme to solve issues on cross-terms and time-frequency resolution. Firstly, we use an Empirical Mode Decomposition (EMD) algorithm reconstructing the collected raw signal to filter out the non-target micro-motion interference and adopt S-transform to improve the time-frequency resolution. Then, a new method of Energy Aggregation is obtained by optimizing the classical S-method, which can suppress cross-terms and background noise. The resolution of the time-frequency distribution obtained after the time-frequency analysis scheme above is sufficient for subsequent feature extraction. Finally, three effective features are extracted, and four motion states are classified by Bagged Trees [25].

The main contributions in this paper can be summarized as follows:

1. This paper proposes a target human motion classification system that can work in the scenarios with non-target micro-motion interference. Under the non-target micro-motion interference caused by a non-target human body, the proposed system can accurately classify four different motion states, including walking without any arm swinging, walking with one arm swinging, walking with both arms swinging, and running.
2. This paper designs a time-frequency analysis scheme, which can (1) filter out non-target micro-motion interference, (2) improve the time-frequency resolution of the collected signal, and (3) suppress the negative influence of cross-terms and background noise.
3. This paper proposes a new method of Energy Aggregation based on the classical S-method. By using S-transform instead of STFT, and increasing the weight of time-frequency point energy spectral density, we greatly improve the time-frequency resolution and mitigate the effect caused by background noise, while maintaining the ability of the S-method in terms of cross-terms suppression and time-frequency aggregation enhancement.
4. This paper uses the software defined radio equipment Universal Software Radio Peripheral (USRP) N210 and GNU Radio software tool-kits [26] to construct the continuous wave radar system. Our experiments show the high performance of the proposed system in terms of its interference resistance and classification accuracy under various experimental scenarios.

## 2. System

### 2.1. System Overview

**Figure 1:**
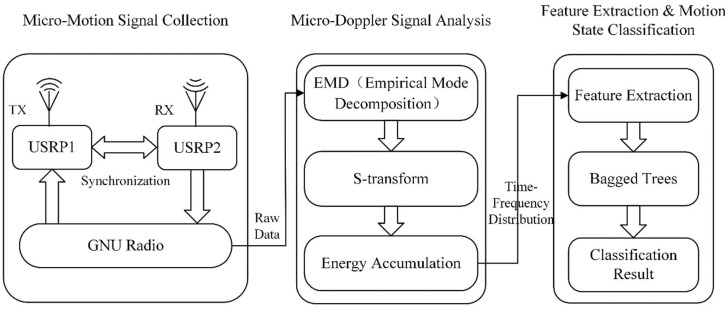
Architecture of the proposed system.

In this paper, we propose a target human motion classification system that can work in the scenarios with non-target micro-motion interference. In the scenarios with non-target micro-motion interference, the proposed system can accurately classify four different human motions, i.e., walking without any arm swinging, walking with one arm swinging, walking with both arms swinging, and running. As shown in Figure 1, the proposed system consists of three parts, as follows: Micro-Motion Signal Collection, Micro-Doppler Signal Analysis, and Feature Extraction and Motion State Classification.

1. Micro-Motion Signal Collection: The micro-motion signals are collected by continuous wave radar transceivers. Such signals are radar echoes and they are represented by discrete complex signals. The transceivers are built on a test-bed using USRP N210 and GNU Radio. USRP N210 is a software-defined-radio equipment, and GNU Radio is an open-source software-defined-radio tool-kit.
2. Micro-Doppler Signal Analysis: We use the micro-Doppler signal analysis module performing time-frequency processing on the raw data, so as to (1) filter out the micro-motion interference generated by the non-target human body, and then (2) improve the time-frequency resolution, (3) suppress the cross-terms and mitigate the effect caused by background noise. In this way, the influence of non-target micro-motion interference on the target human motion can be reduced significantly. Firstly, we use the EMD algorithm to decompose the raw signal into multiple time-frequency components and then select the appropriate components for signal reconstruction, which can filter out the interference. Then, we use S-transform to perform time-frequency analysis on the reconstructed signal. The frequency resolution of the S-transform can be automatically adjusted as the frequency changes, so it can increase the time-frequency resolution of the reconstructed signal. Finally, we use Energy Aggregation to process the signal after the S-transform to suppress cross-terms and mitigate the effect caused by background noise. The signal processed by the above methods of time-frequency analysis reduces the influence of non-target micro-motion interference and generates a high-resolution time-frequency distribution that reflects the real body motion of the target human.
3. Feature Extraction and Motion State Classification: We extract a set of features, i.e., Doppler bandwidth, Micro-Doppler bandwidth and Effective arm-swing time difference, from the time-frequency distribution obtained in the Micro-Doppler Signal Analysis part. Further, we adopt ensemble learning by combining bagging and the decision tree, and accurately classify the four motion states, including walking without any arm swinging, walking with one arm swinging, walking with both arms swinging, and running, under the scenarios with non-target micro-motion interference.

### 2.2. Micro-Motion Signal Collection

**Figure 2:**
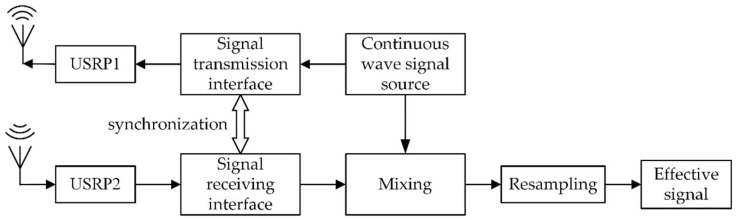
Main components of the continuous-wave radio transceiver.

Continuous wave radar has a simple structure and low requirements on the equipment. Software radio technology has strong flexibility and has great advantages in terms of being open source and low cost. Therefore, we build a continuous-wave radar transceiver using USRP N210 and GNU Radio. Among them, USRP N210 is a software defined radio equipment, and GNU Radio is an open-source software development toolkit. More details about USRP N210 can be found in the Section 3.1 Experiment Testbed. We use such a transceiver as the front-end sub-system for micro-motion signal sensing and collection. The main components of the transceiver are shown in Figure 2.

The continuous wave signal source module generates a digital continuous wave signal. In order to facilitate the analysis of the signal, the discrete continuous wave signal takes the form of a complex signal. In the Field Programmable Gate Array (FPGA) chip of the USRP1 motherboard, the complex signal is up-converted from the digital baseband signal to the intermediate frequency (IF) signal. The digital IF signal is then converted into an analog IF signal by the Digital-to-Analog Converter (DAC) module. Finally, the analog IF signal is up-converted to the analog radio frequency (RF) signal through the RF front end in the USRP1 daughter board, and the antenna completes the transmission of the electromagnetic wave signal. 

The signal received by the antenna passes through the RF front end, the Analog-to-Digital Converter (ADC) module and the FPGA module in the USRP2 sequentially, and finally becomes the digital baseband signal. At the same time, the signal transceiver interface realizes synchronization of continuous wave radar transmission and reception to remove the delay of the device. The transmit and receive signals after synchronization are mixed to filter out the high frequency carrier. Eventually, the signal reduces data redundancy by resampling, and obtains an effective signal for adapting to PC-side data processing.

### 2.3. Micro-Doppler Signal Analysis

The raw data gathered from the micro-motion signal collection module contains non-target micro-motion interference. Also, we use the low-frequency band radar transceiver to collect the human echo signals, so the time-frequency resolution of the raw signal is poor and often accompanied by cross-terms. Thus, this section focuses on removing non-target micro-motion interference, improving time-frequency signal resolution, suppressing cross-terms, and mitigating the effect caused by background noise.

#### 2.3.1. Empirical Mode Decomposition

We use Empirical Mode Decomposition to remove non-target micro-motion interference in the raw data. The EMD is an adaptive time-frequency technique that is well-suited for non-linear and non-stationary time series. One advantage of EMD is that it does not require any prior knowledge of the input signal and is not affected by the selection of a kernel function. Therefore, EMD is widely used in the field of signal processing [27].

EMD decomposes a signal into its Intrinsic Mode Functions (IMFs) based on the time-scale of the oscillations. Each IMF satisfies the following two conditions: (1) the number of extrema and the number of zero crossing must be equal or differ at most by one, and (2) the mean value must be zero. The faster oscillations modes in the signal will occur in the lower-indexed IMFs, whereas the slower oscillation modes will appear in the higher-indexed IMFs. These oscillatory modes are components of the original signal, with the useful feature that each IMF is orthogonal to all of the other IMFs. The orthogonality of the IMFs allows EMD to be used in a number of ways, for example filtering signals, i.e., we can perform signal filtering by selectively adding some of the IMF signals together while omitting others.

The EMD algorithm consists mainly of a sifting process, which is described next. First, we identify the extrema of the signal x(t). Using the extrema, the envelope of the minima, denoted as $X_{min}\left(t\right)$, and the envelope of the maxima, denoted as $X_{max}\left(t\right)$, are formed by using cubic spline interpolation. Next, the average of the upper and lower envelopes is calculated as:

(1) $$X_{env}\left(t\right)=\frac{X_{max}\left(t\right)+X_{min}\left(t\right)}{2},$$

We subtract the average curve $X_{env}\left(t\right)$ from the signal x(t) to get the local oscillation mode $h_{1}\left(t\right)$:

(2) $$h_{1}\left(t\right)=x\left(t\right)-X_{env}\left(t\right),$$

Here, if $h_{1}\left(t\right)$ cannot satisfy the above criteria to be an IMF, we replace $h_{1}\left(t\right)$ with x(t) and repeat the above process until $h_{k}\left(t\right)$ satisfies the criteria, where *k* is the repeating round. If $h_{k}\left(t\right)$ is an IMF, we save it as $C_{1}\left(t\right)$:

(3) $$C_{1}\left(t\right)=h_{k}\left(t\right),$$

We subtract $C_{1}\left(t\right)$ from the signal x(t) to obtain the residue $r_{1}\left(t\right)$, which can be expressed as:

(4) $$r_{1}\left(t\right)=x\left(t\right)-C_{1}\left(t\right),$$

If $r_{1}\left(t\right)$ is not a monotonic function, we repeat the overall procedures by setting $x\left(t\right)=r_{n}\left(t\right)$ and increase *n* by one, where *n* is the repeating round. On the contrary, if $r_{n}\left(t\right)$ is a monotonic function, the process is completed. All IMFs and residue component can be expressed as follows:

(5) $$x\left(t\right)=C_{1}\left(t\right)+C_{2}\left(t\right)+\ldots +C_{n}\left(t\right)+r_{n}\left(t\right),$$

By observing the time-frequency distribution of the raw data, we can find that the time-scale of the oscillation caused by non-target micro-motion interference lies between the target human body swing arm and the target human trunk motion. Therefore, we remove some index IMFs corresponding to non-target micro-motion interference, and then add together the other index IMFs to reconstruct the signal. 

**Figure 3:**
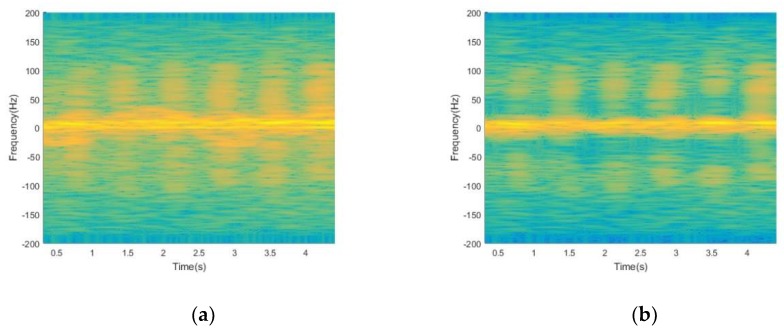
(**a**) Time-frequency distribution of the raw signal before using Empirical Mode Decomposition (EMD) processing; (**b**) time-frequency distribution of the raw signal after using EMD processing.

The time-frequency distributions of the raw signal before and after using EMD processing is shown in Figure 3. As shown in Figure 3a, the interference signal aliases with the signal of target human motions, making the signals hard to use to identify target human motions. It can be seen obviously from Figure 3b that after using EMD processing, most of the non-target micro-motion interferences are filtered out, while other useful components are retained. However, the time-frequency resolution of time-frequency distribution is poor, which is not convenient for subsequent feature extraction. 

#### 2.3.2. S-transform

The S-transform is used to improve signal time-frequency resolution. Extracting pertinent information from noisy, multi-component, and non-stationary signals with complex backgrounds is a challenge in the field of signal processing. The Fourier transform can effectively process time-invariant signals. However, it does not reflect the local time information of the signal, so it has certain limitations on the analysis of time-variant or non-stationary signals. To solve these problems, many time-frequency analysis methods begin to process non-stationary signals in the joint time-frequency domain. Since the micro-Doppler radar echo signal of the human body is a non-stationary signal, we can use the time-frequency analysis method for human-body micro-Doppler signal analysis. 

The S-transform is a linear transform with the advantages of Short-Time Fourier Transform and Wavelet Transform. It uses a variable analyzing window width and preserves the phase information, which allows its frequency resolution to be adjusted automatically as the frequency changes, with high time resolution in the high frequency band and low-frequency resolution in the low-frequency band. Thus, it can overcome the shortcoming of STFT fixed resolution and maintain multi-resolution characteristics [28].

Derived from the STFT, the standard S-transform ST(t,f) of a time varying signal x(t) is given by:

(6) $$ST\left(t,f\right)=\overset{+\infty }{\underset{-\infty }{\int }}x\left(t\right)\omega \left(t-\tau \right)e^{-j2\pi ft}dt,$$

where ω(t) is a Gaussian window centered at t=0 and used to extract a signal segment of x(t). It is defined as:

(7) $$\omega \left(t\right)=\frac{f}{\sqrt{2\pi }}e^{-\;\frac{f^{2}t^{2}}{2}},$$

A constraint is added to restrict the window width σ=1/|f| to be a function of the frequency. The window width *σ* varying inversely with frequency makes the S-transform performing a multi-resolution analysis on the signal. 

Then, the S-transform of the discrete signal x[kT] can be defined as $ST\left[kT,\frac{n}{NT}\right]$:

(8) $$ST\left[kT,\frac{n}{NT}\right]=\overset{N-1}{\underset{m=0}{\sum }}X\left[\frac{m+n}{NT}\right]e^{-\;\frac{2\pi^{2}m^{2}}{n^{2}}}e^{\frac{j2\pi mk}{N}},$$

where *N* is the total number of sampling points and *T* is the time domain sampling interval.

**Figure 4:**
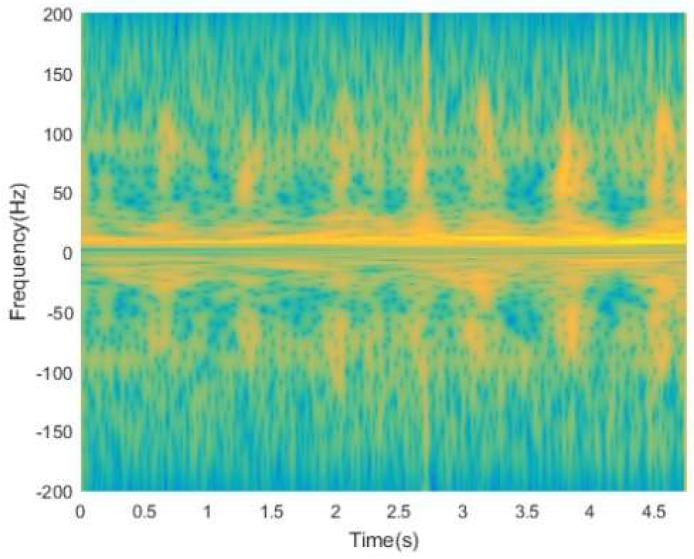
Time-frequency distribution of the signal processed by S-transform.

As shown in Figure 4, the signal processed by S-transform can effectively improve the time-frequency resolution of the signal and maintain multi-resolution. However, the effect caused by cross-terms and background noise is so serious that it is hard to separate the effective signal to extract features.

#### 2.3.3. Energy Aggregation based on S-method

The S-method proposed by Stankovic et al. [29] is widely used in signal processing, which ensures the time-frequency aggregation of multi-component signals and suppresses cross-terms. However, S-method has limited improvement in the time-frequency resolution of the signal. Additionally, when unfiltered non-target micro-motion interference signals appear near the effective signal, which are difficult to filtered, processing the collected signal with S-method can cause the effective signal to be flooded by the background noise signal, thereby making the separation of the effective signal from the collected signal harder. Therefore, we propose a new method based on S-method, named Energy Aggregation, to solve such problems in S-method and maintain its advantages. The details are as below.

Firstly, we briefly summarize the idea of S-method. S-method is proposed to suppress the cross-terms caused by the Winger-Ville distribution. The S-method distribution SM(t,ω) for the continuous signal x(t) is defined as:

(9) $$SM(t,\omega )=\int_{-\infty }^{+\infty }P\left(\theta \right)STFT\left(t,\omega +\theta \right)STFT^{*}\left(t,\omega -\theta \right)d\theta ,$$

where P(θ) is a fixed length window in the frequency domain. Accordingly, S-method distribution for discrete signals is:

(10) $$SM(n,k)=\overset{L}{\underset{l=-L}{\sum }}P\left(l\right)STFT\left(n,k+l\right)STFT^{*}\left(n,k-l\right),$$

where P(l) is a fixed-length window in the frequency domain, and its length is 2L+1. Regarding the S-method distribution, when L=0, the S-method distribution has the distribution characteristics of the STFT, while if L=N/2, it has the distribution characteristics of the pseudo Winger-Ville. Thus, selecting an appropriate window length *L* can simultaneously have the advantages of both, i.e., having a high time-frequency aggregation and suppressing cross-terms. When the frequency domain window P(l) is a rectangular window, Equation (10) can be converted into:

(11) $$SM(n,k)={\left|STFT\left(n,k\right)\right|}^{2}+2Re\overset{L}{\underset{l=1}{\sum }}STFT\left(n,k+l\right)STFT^{*}\left(n,k-l\right),$$

where ${\left|STFT\left(n,k\right)\right|}^{2}$ is the energy spectral density of the time-frequency point (n,k), and the summation is the energy sum of those time-frequency points in the frequency domain window P(l) at the same time point *n*. The basic idea is to supplement the energy of the time-frequency point by adding the energy of the points in the frequency domain window to that of point (n,k). Although the classical S-method has good performance in processing multi-component signals, there are still some shortcomings that cannot be ignored when the signal contains unfiltered interference, including: (1) the STFT in the S-method formula is used to suppress cross-terms. However, its time-frequency resolution is fixed, meaning that the time-frequency resolution of the S-method is poor; (2) when the classical S-method processes the background noise, a large amount of unfiltered interference signal energy is added to the background noise signal. This may likely increase the energy of the background noise signal to a similar extent of the effective signal energy, and then the effective signal might be flooded by the processed background noise signal; (3) the calculation in Equation (11) is somewhat complicated.

To overcome the above three shortcomings, we optimize the classical S-method to obtain a new method: Energy Aggregation. The corresponding improvements are as follows: (1) we replace the STFT in Equation (11) with the S-transform mentioned in Equation (8). The S-transform can overcome the fixed resolution issue of STFT, and meanwhile, it can maintain multi-resolution characteristics of STFT. This allows Energy Aggregation to improve the time-frequency resolution of the signal; (2) we introduce the weight P=2L+1 for ${\left|STFT\left(n,k\right)\right|}^{2}$. This can increase the weight of the time-frequency points’ own energy spectral density in the total energy spectral density and reduce the weight of the supplement energy spectral density. By this way, even if the unfiltered signal energy is added to the background noise, the background noise energy is not to a similar extent of the effective signal energy. Therefore, the difference of energy spectral density between the effective signal and the background noise signal is increased, which can make the effective signal not be flooded by the background noise signal; (3) the second part in Equation (11) is replaced by the calculation of energy spectral density in the proposed Energy Aggregation, as shown in Equation (12) below. This can simplify the calculation. 

The above improvements enable Energy Aggregation to increase the time-frequency resolution of the signal and suppress background noise as well, while maintaining the ability of the S-method in terms of cross-terms suppression and time-frequency aggregation enhancement. The formula for Energy Aggregation EA(n,k) can be expressed as:

(12) $$EA\left(n,k\right)=P*{\left|ST\left(n,k\right)\right|}^{2}+\overset{L}{\underset{l=-L}{\sum }}{\left|ST\left(n,k+l\right)\right|}^{2},$$

where, the first part is the Energy Aggregation calculates the energy spectral density of the time-frequency point (n,k), multiplied by the coefficient *P*. The second part is the sum of the energy spectral density of those frequency points on both sides, with *k* as the axis of symmetry, at the same time point *n*. Since the S-transform has been used in Section 2.3.2, we only need to continue to perform the subsequent calculation of the Energy Aggregation in Equation (12).

**Figure 5:**
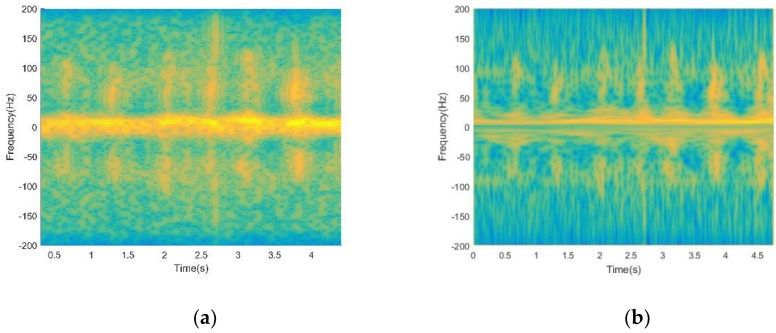
(**a**) Time-frequency distribution of signal processed by S-method; (**b**) time-frequency distribution of signal processed by Energy Aggregation.

The results of the two methods, S-method and Energy Aggregation, are shown in the Figure 5. The results show that although the S-method can improve the time-frequency aggregation and suppress cross-terms, the time-frequency resolution is low and the interference of background noise signals is aggravated, as shown in Figure 5a. Instead, by using the Energy Aggregation proposed in this paper, the time-frequency resolution can be effectively improved, while maintaining the ability of the S-method in terms of cross-terms suppression and time-frequency aggregation enhancement, as shown in Figure 5b. Furthermore, in Figure 5b, the difference of energy spectral density between the effective signal and the background noise signal is more obvious. Thus, the Energy Aggregation obtains the capability of suppressing the background noise and cross-terms, which is more conducive to separating the effective signal for extracting features in the next step.

### 2.4. Feature Extraction and Motion State Classification

#### 2.4.1. Feature Extraction

**Figure 6:**
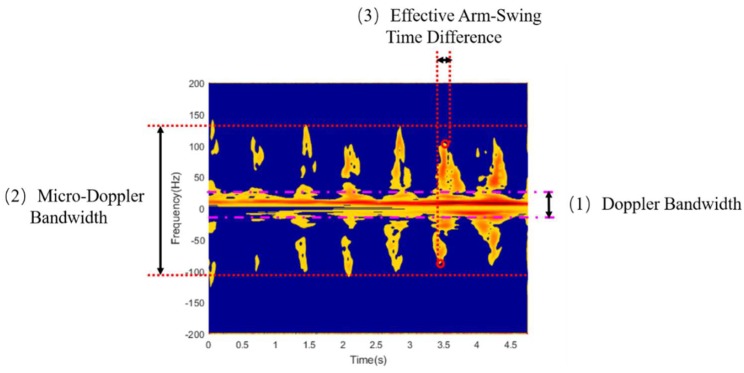
Three features selected from time-frequency distribution.

After Energy Aggregation, we extract three features in the time-frequency distribution, i.e., Doppler Bandwidth, Micro-Doppler Bandwidth, and Effective Arm-Swing Time Difference. The Doppler bandwidth reflects the change in trunk velocity during human motion. The micro-Doppler bandwidth reflects the swinging speed of the arm and the swinging amplitude of the arm during the entire movement. The effective arm-swing time difference reflects the number of swinging arms. As shown in Figure 6, the time-frequency signals are divided by energy gradients. From that we can directly get the Doppler bandwidth and micro-Doppler bandwidth. Then, we extract the peak point of the swing-arm and use the swing-arm matching to obtain the corresponding time and get the effective arm swing time difference. The three features can be summarized as follows:

1. Doppler Bandwidth: This reflects the change of trunk velocity during the movement of the human body. When the value of Doppler bandwidth is small, the movement velocity of the trunk is slow. When it is large, the movement velocity of the trunk is faster.
2. Micro-Doppler Bandwidth: The total bandwidth of the effective signal is used as the micro-Doppler bandwidth, which reflects the situation of the swinging arm. When the value is small, it means that there is no arm swing. When the value is large, the arm swings.
3. Effective Arm-Swing Time Difference: This reflects the time difference between the forward swinging arm and the backward swinging arm, and the number of swinging arms can be decided based on this. A larger value represents an arm-swing, and a smaller value represents two arm-swings.

#### 2.4.2. Motion State Classification

We take the above three features as inputs. Next, we use the idea of ensemble learning to combine bagging and the decision tree, and classify the target human motions under non-target micro-motion interference.

**Figure 7:**
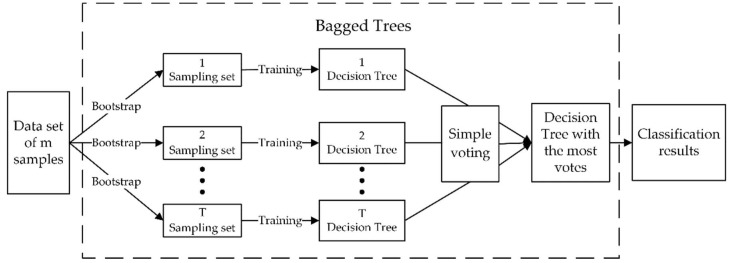
The flow chart of Bagged Trees.

First, given a data set containing *m* samples, we randomly take a sample from the data set into the sampling set and put the sample back into the initial data set. After randomly sampling *m* times, we obtain a sample set containing *m* samples. Then, we repeat the above steps *T* times, and we obtain *T* sampling sets, with each sampling set containing *m* samples. Finally, we train the decision tree based on each sample set to obtain *T* different decision trees, and then use the simple voting method to obtain the decision tree with the highest number of votes. The classification result produced by the decision tree with the highest number of votes is used as the result of Bagged Trees. The flow chart is shown in the Figure 7.

We tested this classification method using ten-fold cross-validation. We divided the data set into ten equal parts, and took nine of them as a training set and used the remainder as a test set. Taking turns in this way, we finally calculated the accuracy of the classification by calculating the mean of each correct rate. Related experimental results can be found in Section 3.2.

## 3. Experimental Evaluation

### 3.1. Experimental Testbed

We built an experimental testbed using software defined radio equipment USRP N210. Specifically, a continuous-wave radar system with a carrier frequency centered at 4.2 GHz is implemented on the USRP N210 device with Sea-based X-band Radar (SBX) daughterboard by using the GNU Radio, an open-source software development toolkit. In this system, a radar transceiver contains two USRPs accompanied by a Multiple Input Multiple Output (MIMO) cable for time synchronization, to implement signal transmission, and for reception, respectively. Here, the SBX daughterboard provides a 40MHz bandwidth and can work at a variety of bands in the 400–4400 MHz range. Two PCB directional RF antennas complete the RF transceiver, whose frequency band is 850–6500 MHz. Also, the GNU Radio is installed in a laptop computer with a Gigabit Ethernet network card. The USRP is connected to the computer by a Gigabit high-speed network cable. The discrete complex signals generated from the USRP are processed in the computer.

**Figure 8:**
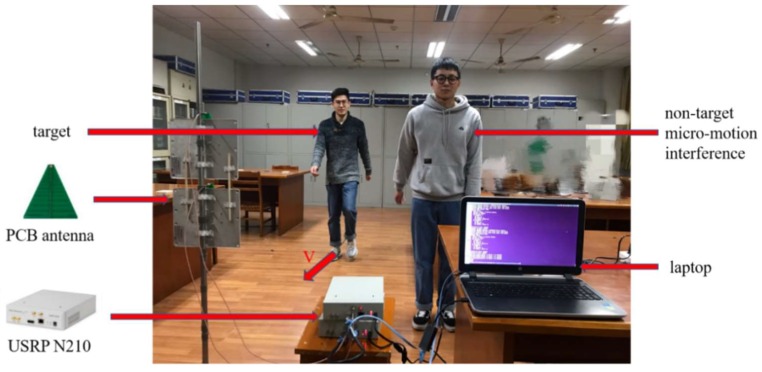
Experimental scene and settings.

The measurement of the data is performed in an indoor environment with the following configuration. The transmitting and receiving antennas are placed under the same vertical plane. The distance between the two PCB antennas is about 10 cm and 1.2 m from the ground. In order to reduce the multipath effect, we place the PCB antenna in the middle position and the target human body moves radially toward the antenna. The range of the measurement is between 0 and 5 m. Non-target human bodies are located 1.5 m away from the antenna and 0.5 m away from the target human. The specific experimental scene is shown in Figure 8. Each acquisition process is 15 s, which is divided into three 5-seconds segments. The first 5 s is static, the middle 5 s is the effective signal of the target human motion, and in the last 5 s the target human body remains in motion. We select the middle 5 s as the valid data segment.

We collect continuous-wave radar echo signals generated from 5 human bodies undergoing different activities, and the signals were collected under the scenarios with non-target micro-motion interference. Only a single target human body is tested at one time, with the target moving directly toward the radar. The signals for a target human body undergoing four different activities are collected, including: (1) walking without any arm swinging (without arm), (2) walking with one arm swinging (single arm), (3) walking with both arms swinging (both arms), and (4) running. Among them, the walking without any arm swinging, the walking with one arm swinging, and the walking with both arms swinging are all maintained at a constant speed, and the running maintains a relatively stable speed. Considering the non-target micro-motion interference, this paper sets two kinds of interference, which are divided into: (1) non-target sudden interference, and (2) non-target continuous swing arm interference. The sudden interference is the sudden swinging or standing for a non-target human body and continuous swing arm interference is when a non-target human body maintains a uniform swinging arm during the whole experimental period.

### 3.2. Results

In the case of non-target sudden interference and non-target continuous swing arm interference, the filtering of the interference and the high resolution of the time-frequency distribution are critical to the system. Next, we show the good processing result of the proposed method on the signal collected by the continuous-wave radar system mentioned above. In addition, the classification results of the four human motion states can also be seen.

**Figure 9:**
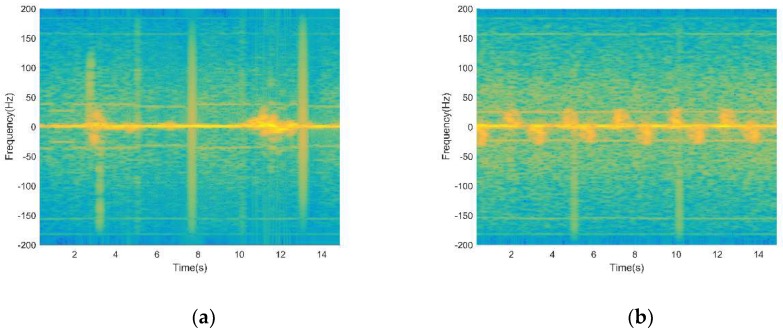
(**a**) Time-frequency distribution of non-target sudden interference; (**b**) Time-frequency distribution of non-target continuous swing arm interference.

Non-target sudden interference is a weak interference. The non-target human body suddenly swings or stands for a shorter duration. The non-target continuous swing arm interference is a strong interference, where the non-target human body maintains a uniform swing arm during the whole experimental period. Time-frequency distribution of non-target micro-motion interference is shown in Figure 9.

**Figure 10:**
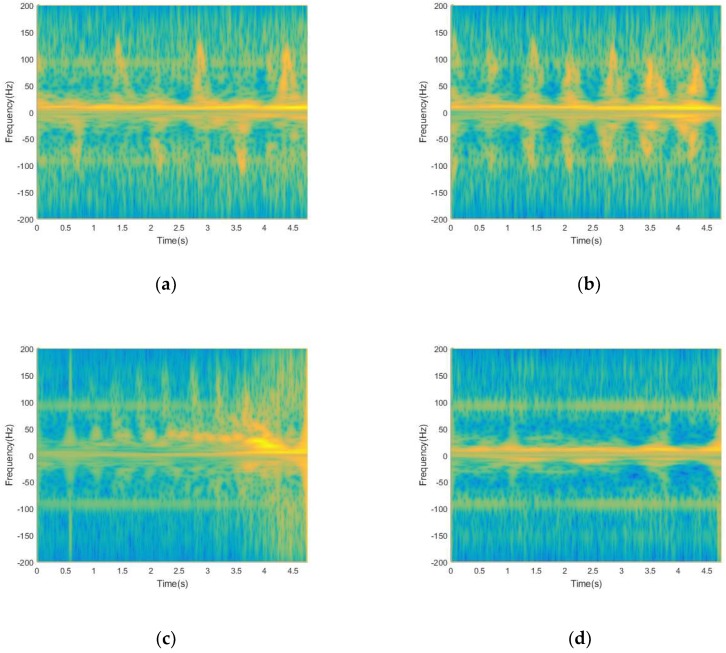
(**a**) Time-frequency distribution of walking with one arm swinging. (**b**) Time-frequency distribution of walking with both arms swinging; (**c**) Time-frequency distribution of running. (**d**) Time-frequency distribution of walking without either arm swinging.

Under the non-target micro-motion interference, walking without any arm swinging, walking with one arm swinging, walking with both arms swinging, and running, the time-frequency distributions of four motion states processed by the time-frequency analysis scheme are shown in the Figure 10:

The collected signal is mainly divided into two parts. One is the torso with stronger energy, and the other is the part around the torso with lower energy. The strongest return in each time-frequency distribution comes from that of the torso, while the periodic micro-Doppler modulations surrounding the torso return come from arm movements. It can be seen from the time-frequency distributions of the four motions that after the signal is processed by the time-frequency analysis scheme, the interference of the non-target micro-motion is reduced, and the background noise and cross-terms caused by the interference are suppressed. Therefore, it is possible to distinguish between the swing arm movement and the trunk movement. At the same time, the time-frequency resolution of the signal is improved, which can reveal the real body of the target human motion. In Figure 10c, the Doppler frequency is highest due to the faster movement of the torso during running. In Figure 10d, there is no swing arm in the walking without any arm swinging, and the micro-Doppler bandwidth is the smallest compared to the other three types of motion. In Figure 10a,b, the effective arm swing time difference of walking with one arm swinging is large, but the time difference of walking with both arms swinging is small. Since the number of swinging arms is different during the two movements, therefore, the time difference of the swing arm pair is different during a complete swing arm process. After feature extraction, we use Bagged Trees to classify human motions and verify the validity of the proposed system with ten-fold cross-validation. The final average classification accuracy rate is 97.3%, and Table 1 give the results of the corresponding confusion matrix.

As can be seen from Table 1, the recognition accuracy rate of running is up to 100%, implying that non-target micro-motion interference has the least impact on it. The accuracy of walking without any arm swinging is 98%, and the accuracy of walking with one arm swinging is 96%. The accuracy of walking with both arms swinging is the lowest, at only 95%. This is because non-target micro-motion interference interferes with the recognition of the effective swing arm pair, which affects the extraction of the time difference. Thus, the walking with both arms swinging is misjudged as walking with one arm swinging.

For non-target sudden interference cases, such as sudden swings, sudden standing, etc., the interference is characterized by non-sustainability and small amplitude. In this case, the target human motion state classification still has a higher classification accuracy, which is basically the same as non-target human body continuous swing arm interference. It can be seen that under the low-frequency radar band in the case of micro-motion interference from non-targets, the proposed system performs well and has high classification accuracy. Also, it can effectively reduce the impact of interference on the target human motion.

### 3.3. Discussion

#### 3.3.1. Comparison with Other Systems

Next, we compare the other three human motion state classification systems with the proposed system in the scenarios with non-target micro-motion interference. In a previous study [30], the authors used S-band continuous wave radar with a carrier frequency of 9.8 GHz to collect target human motion data, and classified the three motion states of walking with no bag, walking with one bag held by one hand, and walking with one bag held by both hands. Firstly, the micro-Doppler signatures are obtained by performing time-frequency analysis on the radar data. Then, two micro-Doppler features are extracted from the time-frequency domain. Finally, the one-versus-one support vector machine is used to realize multi-class classification. We apply the above method to the data obtained in this paper. The results are shown in Table 2. In the case of non-target micro-motion interference, the accuracy rate of this method for the four movement states of the target human body is 88.5%. Among them, accuracy results for walking with one arm swinging and walking with both arms swinging were 78% and 81%, respectively. In scenarios with non-target micro-motion interference, the classification accuracy of these two modes of motion is low, so this system has poor resistance to interference.

In a previous study [24], the authors use Multiwindow AS-method for time-frequency analysis and extract 12 features to classify seven motion states through SVM based on decision tree. Among them, running, walking, walking while holding a stick, and boxing while moving forward are four sports states that are similar to the selected sports types in this paper, so we compare these with this system. We used the same method to classify the four motion states under non-target micro-motion interference. As shown in Table 3, the accuracy is 90.8%. The interference has a great influence on walking with one arm swinging and walking with both arm swinging, and the error rates are 12% and 17%, respectively. Thus, it is easy for misclassification to occur when classifying.

In a previous study [17], a simple feature extraction method is applied to the motion state classification, which is Linear Predictive Coding (LPC). The echo signals are processed using LPC by selecting appropriate sizes for the non-overlapping time window, LPC coefficients, and decision time frames. The confusion matrix is presented in Table 4. When this method is applied to non-target continuous swing arm interference, the accuracy rate of the four motion states is 83.3%. Even worse, the accuracy of walking with one arm swinging and walking with both arms swinging was only 73% and 77%, respectively. Therefore, the influence of interference on this method is more serious.

According to the above results, the classification accuracy rate of the system proposed in previous studies [17,24,30] cannot meet the basic classification requirements in the case of non-target micro-motion interference. However, the system proposed in this paper can resist interference effectively and still have high classification accuracy under interference. To further illustrate the performance of the system, we tested the four motion state classification results in the scenarios without any non-target micro-motion interference. Additionally, we compare the proposed system with the three systems mentioned above in the scenarios with or without non-target micro-motion interference.

**Figure 11:**
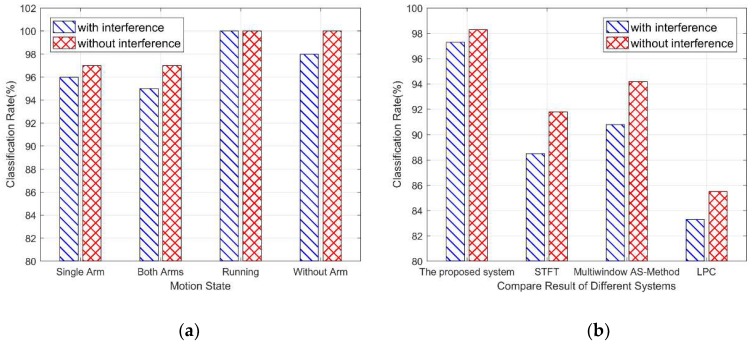
(**a**) Comparison of classification rate of the proposed system with or without interference; (**b**) comparison of classification rate of different systems with or without interference.

As shown in Figure 11a, the system still has a high accuracy rate for the classification of the four motion states in the scenarios without non-target micro-motion interference. Compared with the interference situation, the accuracy rate is slightly improved and the overall stability is stable, indicating that the system has good stability. As shown in Figure 11b, in the scenarios without non-target micro-motion interference, the accuracy of the proposed system, STFT, multi-window AS-method, and LPC are 98.3%, 91.8%, 94.2%, and 85.5%, respectively. The proposed system has a high accuracy rate in the presence or absence of non-target micro-motion interference, and it is slightly improved without interference. However, other systems are more sensitive to interference, and the classification accuracy is significantly improved without interference, which indicates that the proposed system has better robustness and stability.

Additionally, the computational complexity values of the three compared systems are $O\left(Nlog_{2}N\right)$, $O\left(N+Nlog_{2}N\right)$, and $O\left(LP+P^{2}\right)$, and the proposed system has the highest computational complexity, which is $O\left(N+N^{2}log_{2}N\right)$. Although the proposed method has relatively high computation complexity, it can effectively resist interference and improve time-frequency resolution, consequently achieving much better performance, which is more important.

#### 3.3.2. The Classification Accuracy of Different Distances

**Figure 12:**
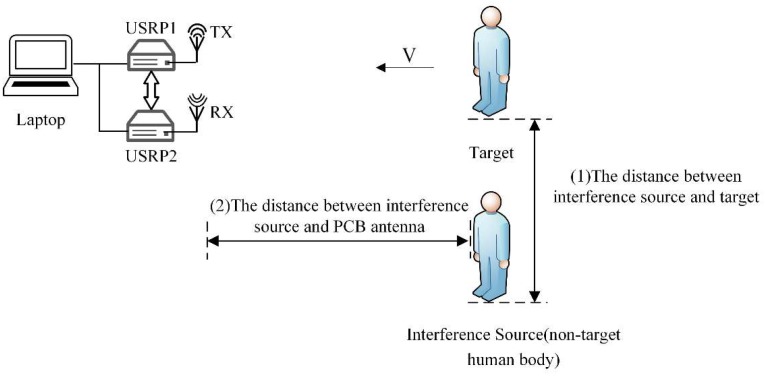
Illustration of two types of distances.

**Figure 13:**
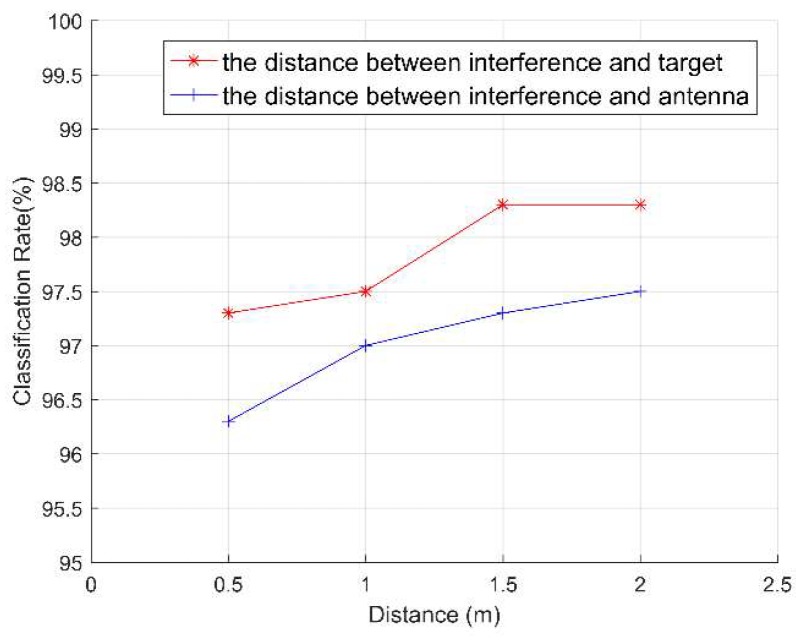
Classification rate of two types of distances.

The distance between the non-target human body (interference source) and the target human body or the PCB antenna affects the classification accuracy of the target human motion state. We conducted a distance experiment to explore this effect. As shown in Figure 12, we define the above two types of distance, including: (1) the distance between interference source and target, and (2) the distance between interference source and the PCB antenna. First, we consider the effect of the distance between interference source and target on the accuracy rate, and set the irrelevant variable, the distance between interference source and PCB antenna, as unchanged. As shown in Figure 13, when the distance between interference source and target is 0.5 m, the interference has the greatest influence on the target human motion. The classification accuracy at this case is 97.3%. As the distance increases, the influence of interference on the target human motion is reduced, so the accuracy is slowly increased. When the distance is 1 m, the classification accuracy is 97.5%. However, when the distance is greater than 1.5m, the interference of non-target micro-motion can be neglected. At this point, the classification accuracy rate is 98.3% which is almost the same as when there is no interference.

Then, we consider the influence of the distance between interference source and PCB antenna on the accuracy, and set the irrelevant variable, the distance between interference source and target, as unchanged. As shown in Figure 13, the classification accuracy is the lowest when the distance between interference source and PCB antenna is 0.5 m, which is 96.3%. As the distance increases, the intensity of the interference signal in the radar echo signal gradually decreases. Therefore, the influence of interference on the target human motion is gradually reduced, and the accuracy of the motion state classification is slowly increased. From the influence of the two interference distances on the classification accuracy of the target human motion, we find that the classification accuracy slowly increases and remains stable as the distance increases. This guarantees the credibility of the system and shows that the system has strong adaptability.

## 4. Conclusions

In the scenarios in which there exist surrounding non-target humans, the interference signal aliases with the signal of target human motion and then generates cross-terms, which make the signal unable to reflect the true motion of the target human. In this paper, we propose a target human motion classification system that can work in environments with non-target micro-motion interference. Under the premise of using low-band radar waves, the proposed system can overcome the non-target micro-motion interference and accurately classify the target human motions. We use EMD and S-transform successively to remove non-target micro-motion interference and improve the time-frequency resolution of the signal. In addition, Energy Aggregation is proposed as a new method by optimizing the classical S-method, which largely reduces the effects of cross-terms and background noise. Then, a set of features are extracted from the time-frequency distribution and are fed in to Bagged Trees for motion classification. Our experiments show that the proposed system can achieve a high accuracy of 97.3% to classify four types of motion states. 

## Figures and Tables

**Table 1:** Confusion matrix results of the proposed system.

	Single Arm	Both Arms	Running	Without Arm
Single Arm	96	3	0	1
Both Arms	3	95	2	0
Running	0	0	100	0
Without Arm	1	1	0	98

**Table 2:** Confusion matrix results of Short-Time Fourier Transform (STFT).

	Single Arm	Both Arms	Running	Without Arm
Single Arm	78	17	0	5
Both Arms	14	81	0	5
Running	0	0	100	0
Without Arm	5	0	0	95

**Table 3:** Confusion matrix results of Multiwindow Adaptive S-method.

	Single Arm	Both Arms	Running	Without Arm
Single Arm	92	8	0	0
Both Arms	12	88	0	0
Running	0	0	100	0
Without Arm	15	2	0	83

**Table 4:** Confusion matrix results of LPC.

	Single Arm	Both Arms	Running	Without Arm
Single Arm	73	20	0	7
Both Arms	23	77	0	0
Running	0	0	100	0
Without Arm	10	7	0	83
